# Distinct Expression Patterns of ICK/MAK/MOK Protein Kinases in the Intestine Implicate Functional Diversity

**DOI:** 10.1371/journal.pone.0079359

**Published:** 2013-11-07

**Authors:** Tufeng Chen, Di Wu, Christopher A. Moskaluk, Zheng Fu

**Affiliations:** 1 Department of Medicine and Digestive Health Research Center, University of Virginia, Charlottesville, Virginia, United States of America; 2 Department of Pathology, University of Virginia, Charlottesville, Virginia, United States of America; 3 Department of Gastrointestinal Surgery, The Third Affiliated Hospital of Sun Yat-sen University, Guangzhou, China; Temple University School of Medicine, United States of America

## Abstract

ICK/MRK (intestinal cell kinase/MAK-related kinase), MAK (male germ cell-associated kinase), and MOK (MAPK/MAK/MRK-overlapping kinase) are closely related serine/threonine protein kinases in the protein kinome. The biological functions and regulatory mechanisms of the ICK/MAK/MOK family are still largely elusive. Despite significant similarities in their catalytic domains, they diverge markedly in the sequence and structural organization of their C-terminal non-catalytic domains, raising the question as to whether they have distinct, overlapping, or redundant biological functions. In order to gain insights into their biological activities and lay a fundamental groundwork for functional studies, we investigated the spatio-temporal distribution patterns and the expression dynamics of ICK/MAK/MOK protein kinases in the intestine. We found that ICK/MAK/MOK proteins display divergent expression patterns along the duodenum-to-colon axis and during postnatal murine development. Furthermore, they are differentially partitioned between intestinal epithelium and mesenchyme. A significant increase in the protein level of ICK, but not MAK, was induced in human primary colon cancer specimens. ICK protein level was up-regulated whereas MOK protein level was down-regulated in mouse intestinal adenomas as compared with their adjacent normal intestinal mucosa. These data suggest distinct roles for ICK/MAK/MOK protein kinases in the regulation of intestinal neoplasia. Taken together, our findings demonstrate that the expressions of ICK/MAK/MOK proteins in the intestinal tract can be differentially and dynamically regulated, implicating a significant functional diversity within this group of protein kinases.

## Introduction

ICK/MRK [1], [2], MAK [3], [4], and MOK [5] are closely related serine/threonine protein kinases, phylogenetically clustered within the CMGC (CDK/MAPK/GSK3/CLK) group of the human kinome [6]. ICK (intestinal cell kinase), named after its cloning origin the intestine [1], is highly conserved and ubiquitously expressed. Human ICK gene produces two transcript variants, both encoding the same protein (632 residues). MAK gene was designated as male germ cell-associated kinase because it is highly enriched in testicular germ cells [3] and specifically expressed in the meiotic phase in spermatogenesis [7]. MAK expression was also reported in respiratory tract, choroid plexus, prostate and retina [4], [8], [9]. MAK gene produces three protein-coding transcripts encoding protein products of 646 residues, 622 residues, and 581 residues respectively [7], [9]. MOK gene is closely related to MAK and ICK/MRK (MAK-related kinase), thus termed as MOK (MAPK/MAK/MRK-overlapping kinase). Human MOK gene has only one transcript encoding a protein product of 419 residues. MOK mRNA was detected in mouse intestine, brain and lung, and highly enriched in testis and kidney [5], [10].

ICK/MAK/MOK proteins share significant sequence and structural homology to both MAPKs (mitogen-activated protein kinases) and CDKs (cyclin-dependent protein kinases) in the catalytic domain. They all contain a MAPK-like TXY motif in the activation loop that is essential for their full activation [1], [3], [5], [11], [12]. However, they diverge significantly in the composition of their C-terminal non-catalytic domains which may determine their signaling specificities. The regulatory mechanisms of ICK/MAK/MOK appear to be very different from that of MAPKs in that they are not acutely activated by growth factors [1], [5]. The upstream activating kinase for ICK and MAK is CCRK (cell cycle-related kinase), not MEKs (the upstream activating kinases for MAPKs) or CDK7 (the upstream activating kinase for CDKs) [12], [13]. Recently, the biological functions of ICK and MAK have been linked to human diseases. A recessive loss-of-function point mutation R272Q of ICK was identified as the causative mutation in a neonatal lethal multiplex human syndrome ECO (endocrine-cerebro-osteodysplasia) [14]. Multiple point mutations in the kinase domain of MAK or an Alu-insertion in exon 9 of MAK have been suggested as potential causes of retinitis pigmentosa, a genetically heritable and autosomal recessive disease [15], [16].

In the intestine, ICK and MOK messages specifically localize to the crypt region [1], [10]. Knockdown of ICK expression in intestinal epithelial cells *in vitro* led to a significant decrease in cell proliferation, implicating a pro-proliferation role for ICK in the intestine [17]. In HT-29 cells, which closely resemble undifferentiated intestinal crypt cells, MOK activity was induced by either sodium butyrate treatment or ectopic expression of Cdx2, which are known to inhibit growth and induce differentiation [10]. This observation suggests a possible role for MOK in the regulation of intestinal epithelial differentiation. Despite the functional implications from these *in vitro* data, the biological functions of ICK/MAK/MOK proteins in the intestine are still elusive due to the lack of *in vivo* evidence from either gain-of-function or loss-of-function studies. Since the expression and distribution pattern of a gene may provide some critical insights into its biological functions, in this report we attempted to assess these descriptive but essential features of the ICK/MAK/MOK family using intestine as the model system. We have demonstrated that ICK/MAK/MOK proteins display distinct expression patterns along the duodenum-to-colon axis in the intestine during postnatal development. More importantly, over-expression of ICK, but not MAK, in primary human colon cancer specimens and a significant up-regulation of ICK protein in parallel with a marked down-regulation of MOK protein in mouse intestinal adenomas implicate distinct roles for ICK/MAK/MOK proteins in the initiation and development of intestinal cancer.

## Materials and Methods

### Animals and Ethics Statement

All animal experiments were carried out according to NIH Animal Welfare Guidelines after approval by the University of Virginia School of Medicine Institutional Animal Care and Use Committee (protocol number 3480). Young adult male and female wild-type mice C57BL/6J were purchased from The Jackson Lab (Bar Harbor, ME) as breeding pairs.

### Antibodies

A rabbit polyclonal antibody was raised against the C-terminal peptide (residues 388-400) of mouse ICK, as described in [17]. A rabbit polyclonal antibody raised against the C-terminal peptide (residues 603-622) of mouse MAK was a generous gift from Professor Masabumi Shibuya (University of Tokyo), as described in [3], [7]. A rabbit polyclonal antibody was raised against the C-terminal peptide (residues 325-341) of mouse MOK and used in this study (Genscript). Antibodies to Bmi1, FoxA2, E-Cadherin, Vimentin, Cdx2, β-Actin, Akt/PKB, and ERK1/2 were all from Cell Signaling Technology. Anti-GPCR GPR49 (Lgr5) rabbit monoclonal antibody (clone EPR3065Y) was from Abcam. Anti-αSMA (Smooth Muscle Actin) rabbit polyclonal antibody was from eBioscience. ERK2 (D-2) mouse monoclonal antibody were from Santa Cruz Biotechnology. Monoclonal anti-PCNA (clone PC10) antibody was from Sigma.

### Tissue harvest and extract preparation

Snap-frozen tissues dissected from 8 week-old young adult C57BL/6J mice were purchased from The Jackson Lab. Also from The Jackson Lab were frozen intestinal adenomatous polyps and their adjacent normal intestinal tissues from the C57BL/6J-APC^min/J^ mice. For postnatal development study, offspring were euthanized on postnatal days (p) 1, 4, 7, 14, and 21. Each time point is represented by four mice. For spatial analysis of gene expression along the duodenum-to-colon axis, the mouse intestinal tube was regionally divided after rapid dissection as previously described [18]. Tissues were snap-frozen in liquid nitrogen and stored at -80°C until use. Frozen tissues were grinded into fine powders on dry-ice and lysed in RIPA buffer (20 mM Tris, pH 7.5, 150 mM NaCl, 1% Nonidet P-40, 0.5% sodium deoxycholate, 1 mM EDTA, 0.1% SDS) containing protease inhibitors cocktail (Roche) and phosphatase inhibitors (1 mM sodium orthovanadate, 5 mM sodium fluoride, 1 µM microcystin LR, and 5 mM β-glycerophosphate). Tissue lysates were cleared by centrifugation, and the supernatant was then saved and stored frozen.

### Isolation of intestinal epithelial and mesenchymal cells

A modified version of the intestinal epithelial cell isolation protocol from [19] was used. Small intestine and colon were dissected out, removed of mesentery/fat tissues, cut open longitudinally to remove feces, and rinsed extensively with cold 1X PBS (without Ca^+2^ and Mg^+2^) before being cut into 1-cm pieces. Small pieces of the intestinal tissue were incubated with very gentle agitation in cold 1X PBS (without Ca^+2^ and Mg^+2^) containing 5 mM EDTA, 0.1 mM DTT, and 15 mM HEPES at 4°C for 45 min. Tissue pieces were gently rinsed with 1X PBS followed by rigorous shaking for 15 seconds in 10 ml PBS to dislodge villi/crypts. The PBS containing detached villi/crypts was then transferred to a 50 ml conical tube. This shaking/dislodging step was repeated 3-4 times until there was a visible and dramatic reduction in the yield of detached villi/crypts observed under a light microscope. The villi/crypts obtained from these shakings were pooled immediately and recovered by gentle centrifugation for 5 min followed by multiple gentle rinses. The epithelial cells (isolated villi and crypts) and the mesenchymal cells (rest of the intestinal tissue) were homogenized in RIPA lysis buffer containing protease and phosphatase inhibitors. Epithelial and mesenchymal cell markers (E-Cadherin, Vimentin, and Smooth Muscle Actin) were used to assess the specificity of isolated cell fractions.

### Western blot

Tissue or cell extracts were mixed with an equal volume of 2X Laemmli sample buffer (120 mM Tris-HCl, pH 6.8, 4% SDS, 20% glycerol, 10% β-mercaptoethanol, 0.02% bromophenol blue), boiled for 5 min, loaded on a SDS-gel, and then transferred to a PVDF membrane for Western blotting. Briefly, the membrane was blocked for one hour in 5% dry milk, and then incubated with primary antibody (1–2 µg) in TBS containing 0.1% Tween-20 and 5% bovine serum albumin (BSA) for 90 min at room temperature or overnight at 4°C followed by extensive rinses and one-hour incubation with horseradish peroxidase (HRP)-conjugated secondary antibody (1∶10,000 dilution). After extensive rinses, chemiluminescence signals were developed using Millipore Immobilon ECL reagents.

### Human colon cancer specimen

Matched colonic adenocarcinoma and non-neoplastic colonic mucosa frozen tissue samples were obtained from the tissue bank of the University of Virginia Biorepository and Tissue Research Facility, previously procured with Institutional Review Board (IRB) approval and used for this project with additional IRB review and approval. The tissue was embedded in OCT compound and subjected to frozen histologic sectioning on a cryostat. The histologic sections were used to dissect areas from the frozen tissue samples corresponding to greater than 70% epithelial cells from neoplastic and non-neoplastic areas, respectively.

### Statistical analysis

Quantification results from nine pairs of human colon cancer tumor tissues and their adjacent normal tissues were analyzed by Paired-sample *t*-test. Data were presented as means ± standard error. P-values were two-sided and less than 0.05 were considered as significant.

## Results

### Ubiquitous expression of ICK protein versus restricted expression of MAK and MOK proteins in both young adult and embryonic mouse tissues

**Figure 1:**
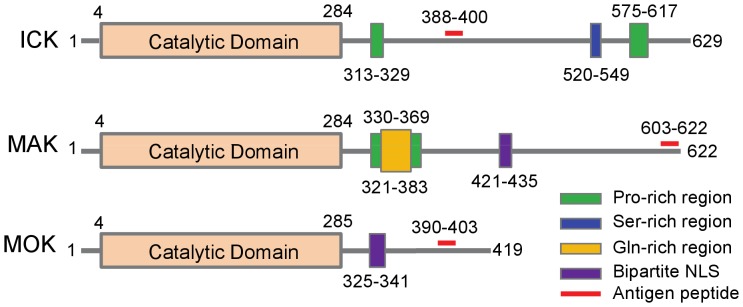
Schematic illustration of the structural domains and features of murine ICK, MAK and MOK protein kinases. Although ICK, MAK and MOK protein kinases are very similar in their N-terminal catalytic domains, they differ markedly in the structural organization and features in their C-terminal non-catalytic domains. Note that the antigen peptides for ICK, MAK, and MOK proteins are all located at the C-terminal domain by design so as to ensure the antibody specificity.

Given that ICK, MAK and MOK proteins are very similar in their N-terminal catalytic domains but differ significantly in their C-terminal non-catalytic tail domains, rabbit polyclonal antibodies were raised against peptide sequences that are located in their respective C-terminal domains in order to ensure the antigen specificity, as indicated in Figure 1. The antigen specificities for ICK and MAK antibodies used in this study have been previously confirmed [7], [17], [20]. We hereby confirmed the specificities of ICK, MAK and MOK antibodies in gastrointestinal cells by assessing target short-hairpin RNA interference (shRNA)-mediated knockdown effects on their protein levels on Western blot (Fig. S1).

**Figure 2:**
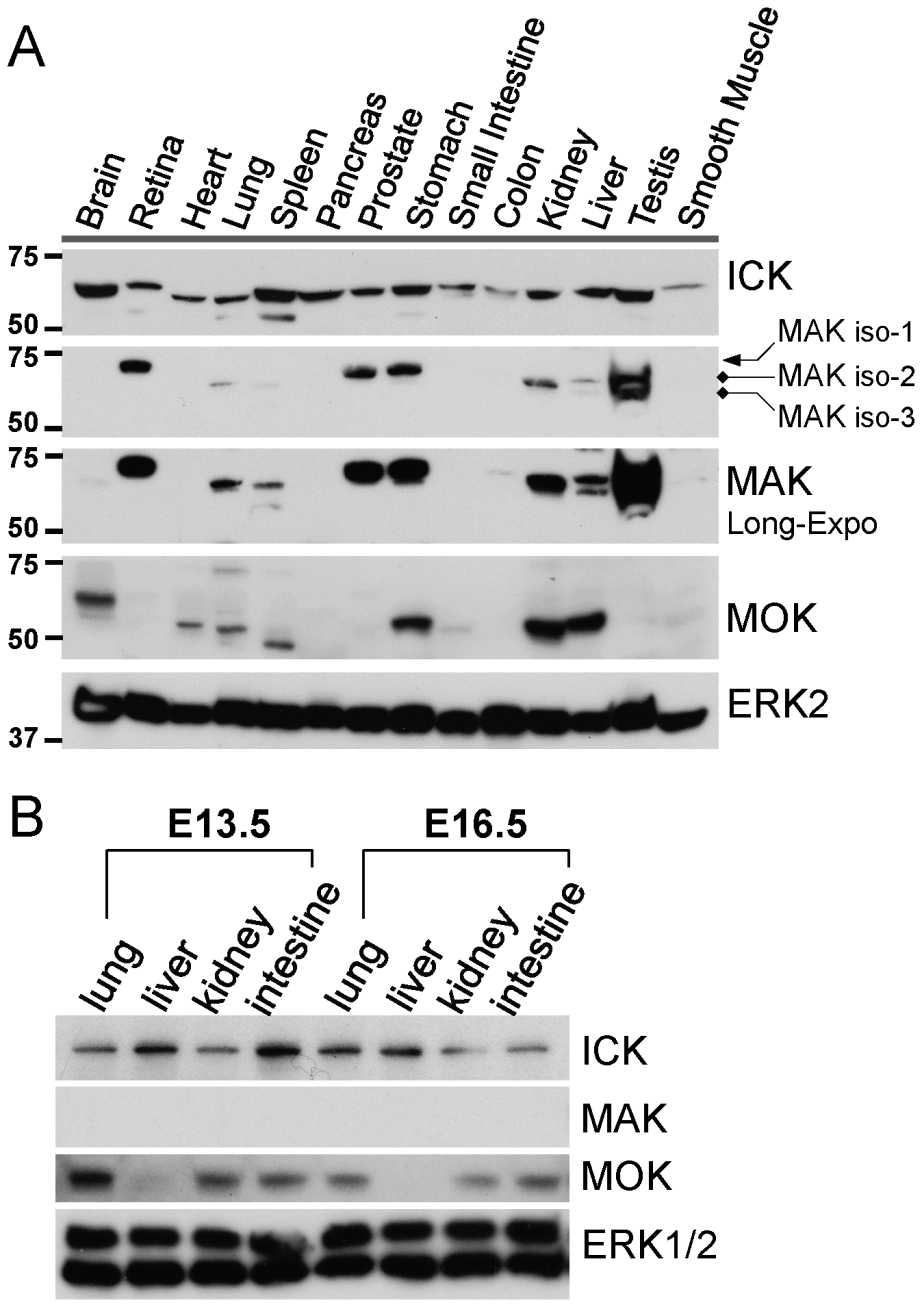
Expression of ICK/MAK/MOK proteins in young adult and embryonic mouse tissues. (A) Equal amount of total proteins (50 µg) extracted from various frozen tissues of young adult C57BL/6J mice (8 week-old) were Western blotted against antibodies recognizing ICK, MAK, and MOK respectively. Western blot signal of ERK2, closely related to ICK/MAK/MOK, was also shown as a control to indicate a universal expression pattern in various young adult mouse tissues. (B) Equal amount of total proteins (50 µg) extracted from frozen tissues (lung, liver, kidney and intestine) of mouse embryos of E13.5 and E16.5 were Western blotted against antibodies recognizing ICK, MAK, and MOK respectively. Western blot signals of ERK1/2 were shown as controls to indicate a universal expression pattern in various mouse embryonic tissues.

Using these affinity-purified rabbit polyclonal antibodies, we first analyzed the expression profiles and relative abundances of ICK, MAK and MOK proteins in various young adult and embryonic mouse tissues (Fig. 2 and Fig. S3). Consistent with the ubiquitous expression pattern of its mRNA [1], [2], ICK protein was widely expressed in either young adult (Fig. 2A) or embryonic (Fig. 2B) mouse tissues examined. In comparison, the expression profiles of both MAK and MOK proteins were more restricted. In agreement with its mRNA distribution pattern [3], MAK proteins were predominantly enriched in testis. However, we also observed the strong expression of MAK proteins in retina, prostate, stomach, and kidney, as well as the relatively weak expression of MAK proteins in liver, lung, spleen, and colon of young adult mice. MAK protein signal was not detected in lung, liver, kidney, and intestine from mouse embryos of E13.5 or E16.5, suggesting that MAK proteins only participate in certain biological activities in these organs at post natal stages. In young adult mice, MOK protein (∼50 KD) was strongly expressed in kidney, liver, stomach and weakly expressed in heart, lung, spleen and small intestine. A larger protein species (∼60 KD) reactive to the MOK antibody was also detected in brain, which may represent a MOK product resulted from post-translational modification or gene hybridization. At embryonic stages E13.5 and E15.5, MOK protein (∼50 KD) was detected in kidney, lung and intestine, but not in liver. These results demonstrate that although MAK and MOK display a more restricted tissue expression pattern than that of ICK, they may play an important role in the development and function of multiple organ systems as well.

### Regional expression patterns of ICK/MAK/MOK proteins along the duodenum-to-colon axis in the intestine

**Figure 3:**
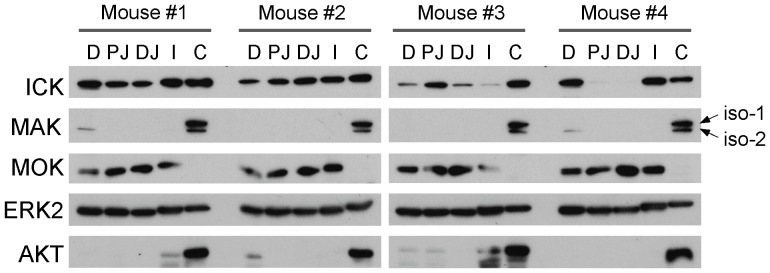
Expression of ICK/MAK/MOK proteins along the duodenum-to-colon axis in the intestine. The intestinal tube was rapidly dissected from four littermates of postnatal day 21 and segmented into duodenum (D), proximal jejunum (PJ), distal jejunum (PJ), ileum (I), and colon (C) respectively. Tissue extracts were prepared from frozen intestinal tissues. Equal amount of total proteins (50 µg) were loaded on Western blot against ICK, MAK, and MOK antibodies respectively. ERK signal was shown to demonstrate an equal distribution pattern along the duodenum-to-colon axis, whereas AKT signal was shown to demonstrate a colon-enriched distribution pattern under the normal steady-state condition.

Very little is known about how ICK, MAK, and MOK proteins are spatially distributed in the intestine. Since their antibodies have not been proven to be suitable for applications in immunohistochemistry and immunofluorescence, we chose to analyze the regional expression patterns of ICK/MAK/MOK proteins along the duodenum-to-colon axis by Western blot. Equal amount of total proteins extracted from duodenum, proximal jejunum, distal jejunum, ileum, and colon tissues were subjected to Western blot analysis (Fig. 3). Overall, ICK protein is ubiquitously expressed along the duodenum-to-colon axis, albeit with varied levels of expression between different segments. MAK gene encodes three different isoforms, two of which were detected in the intestine by Western blot (Fig. S2). MAK isoform-1 (MAK-1) and isoform-2 (MAK-2) proteins were primarily expressed in colon, while MAK-2 was barely detectable in duodenum of the small intestine. Although MOK proteins appeared to be ubiquitously expressed and evenly distributed in different segments of the small intestine, no MOK signal was detected in colon. In comparison, ERK2 signal displayed a ubiquitous and uniform expression pattern along the duodenum-to-colon axis whereas Akt/PKB signal was predominantly localized in colon under the steady-state condition.

### Postnatal developmental expression patterns of intestinal ICK/MAK/MOK proteins

**Figure 4:**
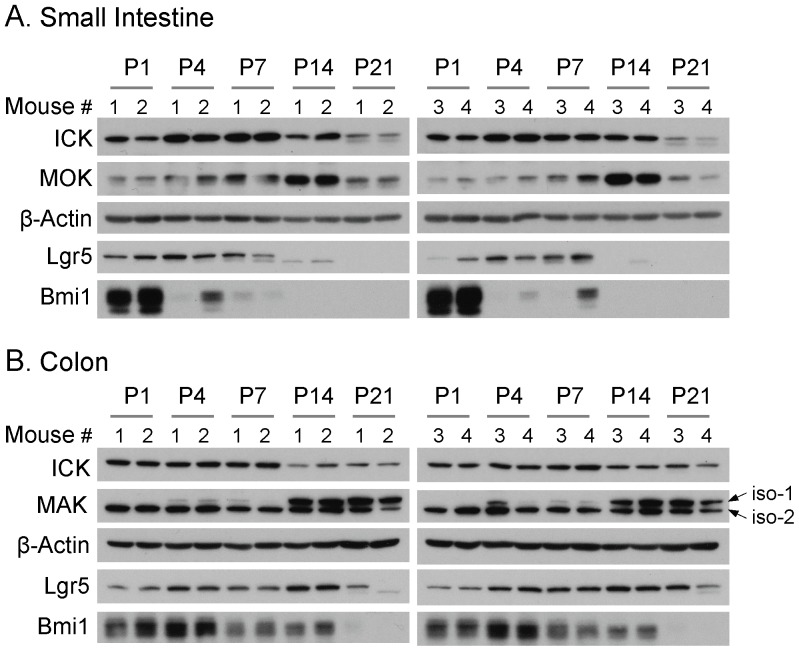
Expression of ICK/MAK/MOK proteins in the mouse intestine during postnatal development. The small intestine or colon was rapidly dissected from four littermates of postnatal day (P) p1, p4, p7, p14, and p21. Tissue extracts were prepared from frozen intestinal tissues. Equal amount of total proteins (50 µg) were loaded on Western blot against ICK, MAK, and MOK antibodies respectively. The β-Actin signal was shown on Western blot to indicate equal loading of total proteins.

We next analyzed the expression profiles of ICK/MAK/MOK proteins in small intestine and colon during postnatal development (Fig. 4). Equal amount of total proteins extracted from the small intestine and colon tissues of mice at postnatal day 1 to day 21 were applied to Western blot against ICK, MAK, and MOK antibodies, respectively. ICK proteins appeared to be consistently expressed during postnatal development until a decline in the expression level occurred in small intestine around postnatal day 21. This decline in ICK protein level also occurred in colon, albeit to a much lesser degree and at an earlier time point (postnatal day 14). Interestingly, MOK protein in small intestine displayed a sharp peak in the expression level at postnatal day 14. In comparison to ICK and MOK, MAK proteins exhibited isoform-specific expression profiles in colon during postnatal development. On one hand, MAK-2 proteins showed a consistent level of expression throughout the postnatal stages. On the other hand, MAK-1 proteins were not detectable at early postnatal stages until postnatal day 4, and the peak level of expression was reached around postnatal day 14. These data together have demonstrated that ICK/MAK/MOK proteins exhibit divergent expression patterns during the postnatal intestinal development.

Two functionally distinct populations of intestinal stem cells exist in the crypt region [21], the mitotically active stem cells marked by Lgr5 [22] and the quiescent stem cells marked by Bmi1 [23]. Since both ICK and MOK messages localize to the intestinal crypt, it has been speculated that the expressions and functions of ICK and MOK proteins may be associated with the intestinal stem cell activities [1], [10]. To assess whether there is any correlation between the expression patterns of ICK and MOK proteins and the intestinal stem cell markers, we analyzed the expression of intestinal stem cell markers Lgr5 and Bmi1 proteins during postnatal development in the intestine. Our results indicate that Lgr5 protein was highly expressed from postnatal day 1 to day 7 in small intestine, and a significant decline in the expression level of Lgr5 protein occurred by postnatal day 14. In comparison, the level of Bmi1 protein peaked at postnatal day 1 and drastically declined by postnatal day 4 in small intestine. Similar results were obtained from colon, except that both the peak and the decline in either Lgr5 or Bmi1 protein levels occurred at a later postnatal development time point. In small intestine, the expression pattern of ICK protein partially overlapped with that of Lgr5 or Bmi1 through early stages of postnatal development. In comparison, Lgr5 and Bmi1 proteins were weakly or barely detected at postnatal day 14 in small intestine when the peak of MOK protein was observed, suggesting very little correlation between the expression of MOK protein and intestinal stem cell activities during postnatal development.

### Differential partition of ICK/MAK/MOK proteins between intestinal epithelium and mesenchyme

**Figure 5:**
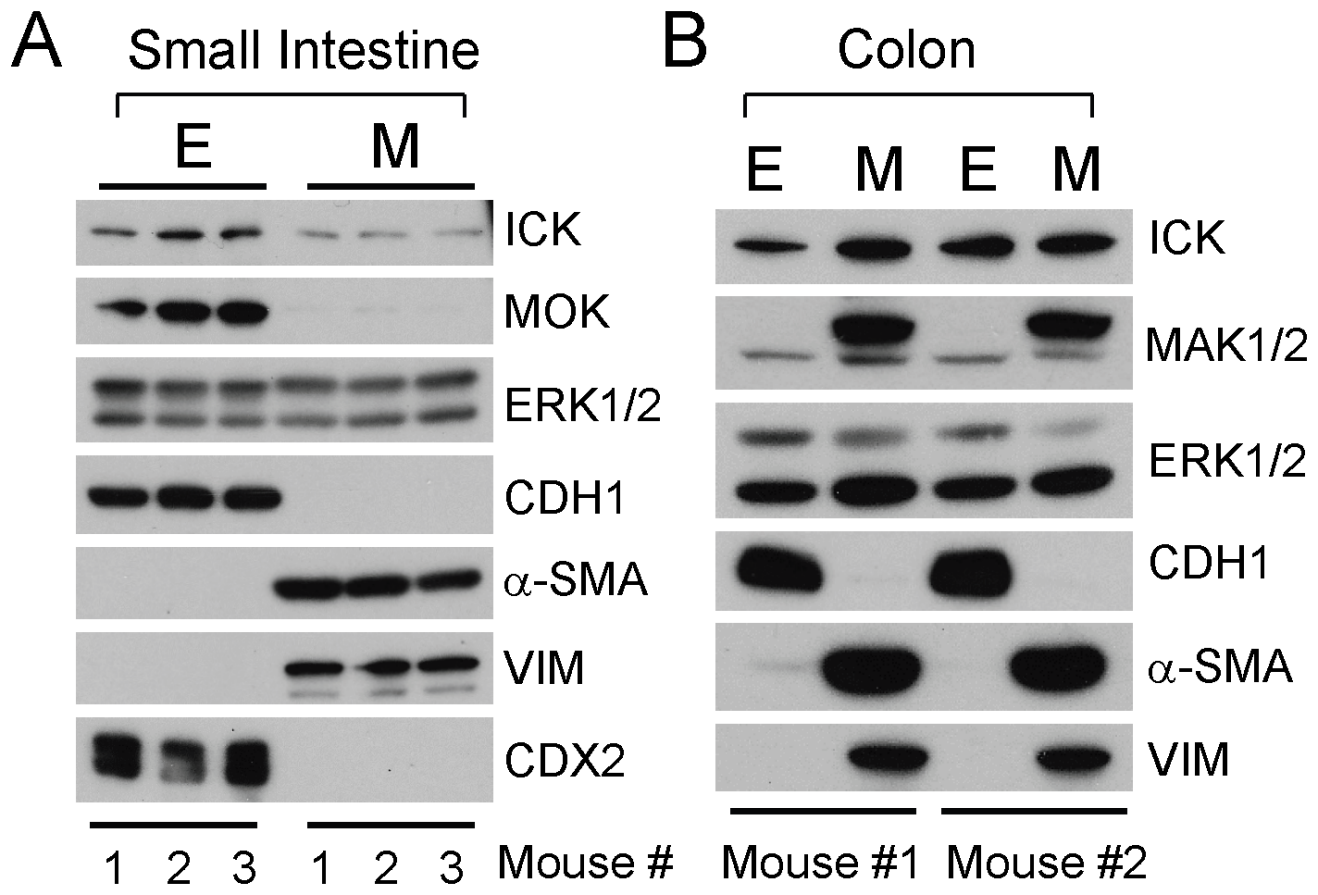
Differential partition of ICK/MAK/MOK proteins between intestinal epithelial and mesenchymal cells. Intestinal epithelial cells and mesenchymal cells were isolated and enriched from small intestine (A) and colon (B) tissues of young adult C57BL/6J mice. ICK/MAK/MOK protein signals in these two distinct cell populations were analyzed on Western blot. The E-Cadherin signal was used as a marker for epithelial cells and the VIM (Vimentin) and α-SMA (α-Smooth Muscle Actin) signals were used as markers for mesenchymal cells. The ERK1/2 signals were shown to indicate an equal distribution pattern between the epithelial and mesenchymal cell populations.

We further analyzed the tissue-distribution specificities of ICK/MAK/MOK proteins in the intestine. Using a conventional intestinal epithelial cell isolation method, we isolated and enriched both intestinal epithelial and mesenchymal cell populations, as confirmed by the restricted expression of epithelial cell marker E-cadherin (CDH1) and mesenchymal cell markers vimentin (VIM) and smooth muscle actin (α-SMA) on Western blot (Fig. 5). It is evident that in small intestine and colon both epithelial and mesenchymal cells expressed ICK proteins. In contrast, MOK protein was restricted to the intestinal epithelial cells (Fig 5A), consistent with the epithelial expression of its upstream transcription regulator CDX2 [10]. Intriguingly, MAK proteins exhibited isoform-specific distribution patterns in colon (Fig 5B). MAK-1 was strictly restricted to the mesenchymal cell population whereas MAK-2 was present in both epithelial and mesenchymal cells, suggesting the two MAK isoforms in colon may be required for different biological functions.

### Over-expression of ICK, but not MAK, in human primary colon cancer specimens

**Figure 6:**
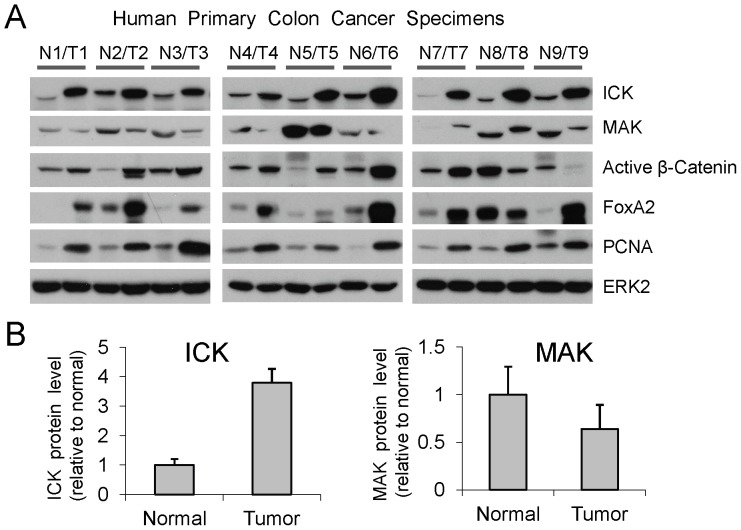
ICK and MAK proteins exhibited distinct expression patterns in human colorectal tumors. (A) Tissue extracts from nine pairs of human specimens macro-dissected from primary colon tumor (T) and its surrounding normal mucosa (N) were prepared. Equal amount of total proteins (30 µg) from tissue extracts were loaded for Western blot. ERK2 signals indicate equal loading of total proteins. PCNA was used as a proliferation marker. (B) Western blot signals of ICK and MAK were quantified by densitometry and normalized against ERK2 signals. ICK and MAK protein levels in tumors relative to their adjacent normal tissues were shown as mean ± SE, n = 9, P<0.05, Paired *t*-Test.

Given that ICK and MAK are closely related and they support proliferation *in vitro* [8], [9], we speculated that over-expression of ICK and/or MAK may correlate closely with malignant proliferation in the colonic epithelium. Thus, we analyzed the levels of ICK and MAK proteins in a panel of human primary colon cancer specimens paired with their adjacent normal colonic mucosa tissues. ICK protein level was significantly elevated, albeit to various degrees, in all of the nine tumor tissues as compared with their adjacent normal tissues (Fig. 6). To our surprise, MAK protein levels in the majority of tumor tissues were either lower than or similar to those in the matched normal tissues. These results clearly demonstrate that the levels of ICK and MAK proteins can be differentially regulated in colon cancer, implicating distinct functional outputs from ICK and MAK in the intestine.

Both β-Catenin and FoxA2 are capable of strongly activating the ICK promoter activity *in vitro* [24]. Accumulation of nuclear β-Catenin (the active β-Catenin) is the leading cause for the initiation of intestinal cancer [25]. Over-expression of FoxA2 has been linked to colon cancer development and progression [26], [27]. Therefore, we also examined the expression of these two transcription factors in this panel of colon cancer specimens. Seven out of nine colon tumors exhibited elevated expression of both active β-Catenin and FoxA2 proteins as compared with their paired normal tissues. In tumor T9, active β-Catenin protein was down-regulated but FoxA2 protein was up-regulated as compared with its paired normal control N9. In tumor T8, both transcription factors were down-regulated as compared with its paired normal control N8. These results demonstrate that the majority of colon tumors examined displayed elevated expression of active β-Catenin and/or FoxA2, providing a possible mechanism underlying ICK over-expression in colon cancer. However, activation of other transcription factors may also be involved in the up-regulation of ICK expression in a small subset of colorectal tumors.

### Up-regulation of ICK concomitant with down-regulation of MOK in intestinal adenomas

**Figure 7:**
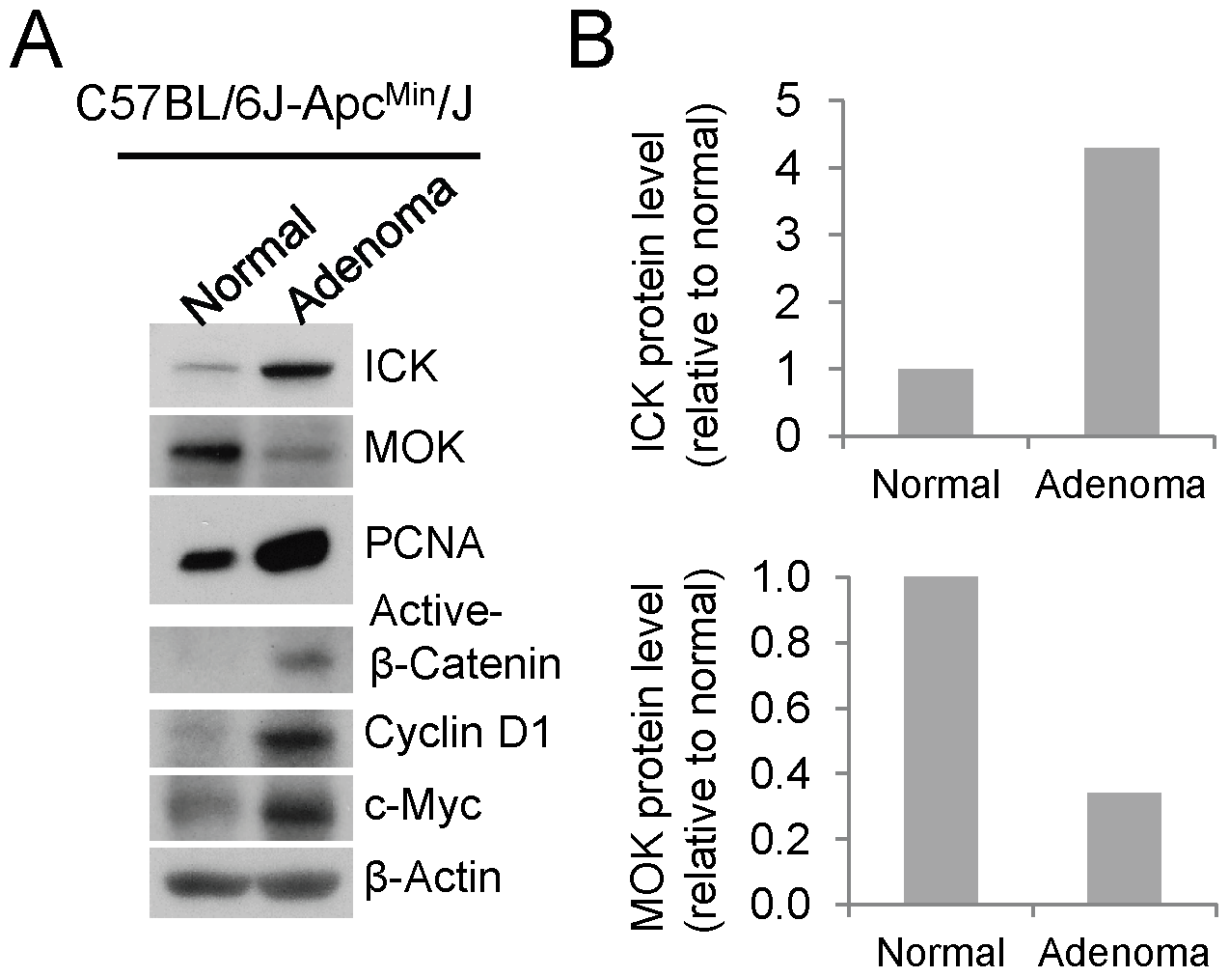
ICK and MOK proteins displayed opposite expression patterns in mouse intestinal adenomas. (A) Tissue specimens macro-dissected from intestinal adenomatous polyps and their adjacent normal mucosa were obtained from C57BL/6J-Apc^min^/J mice (The Jackson Lab). Equal amount of total proteins (30 µg) from tissue extracts were Western blotted against ICK and MOK antibodies respectively. PCNA signal was shown as a proliferation marker to indicate malignant proliferation in the intestinal adenomas. Western signal of β-Actin was shown to indicate equal loading of total proteins. (B) Western blot signals of ICK and MOK were quantified by densitometry and normalized against the β-Actin signal. Shown here were the protein levels of ICK and MAK in intestinal adenomas relative to their adjacent normal tissues.

Our *in vitro* knockdown study has demonstrated an important role for ICK in promoting intestinal epithelial cell proliferation and cell cycle progression [17]. Although there is a lack of direct evidence to indicate the biological functions of MOK in the gastrointestinal tract, an elegant *in vitro* study in HT-29 cells has provided some indirect evidence supporting a role for MOK in the regulation of intestinal epithelial cell differentiation [10]. Based on these observations, we hypothesized that ICK and MOK may play opposite roles in regulating proliferation and differentiation of intestinal epithelial cells *in vivo*. This hypothesis motivated us to assess the protein levels of ICK and MOK in the intestinal adenomatous polyps as compared with their adjacent normal mucosal tissues in the small intestine of the APC/Min mice, a well-established mouse model of intestinal tumorigenesis [28]. Min (multiple intestinal neoplasia) is a mutant allele of the murine *Apc* (*adenomatous polyposis coli*) locus, encoding a nonsense mutation at codon 850. The Min mouse can develop up to 100 adenomatous polyps (adenomas) in the small intestine, which are primary precursor lesion for the development of most colon carcinomas. In the intestinal adenomas where intestinal epithelial cells undergo malignant proliferation as indicated by up-regulation of the proliferation marker PCNA, ICK protein level was significantly elevated whereas MOK protein level was dramatically reduced relative to the adjacent normal tissues, in concomitant with activation of the Wnt/β-Catenin pathway as indicated by up-regulation of the active β-Catenin and its downstream targets Cyclin D1 and c-Myc (Fig. 7). This result indicates that the expression levels of ICK and MOK proteins are regulated in opposite directions during the formation of intestinal adenomas, providing a strong argument that ICK and MOK may perform opposite biological functions in the intestinal epithelium.

## Discussion

The location and the dynamic expression pattern of a signaling protein are pivotal factors in determining its biological effects. In this report, we examined these essential features of a group of closely related serine/threonine protein kinases ICK/MAK/MOK in the intestine in order to gain some novel insights into their biological activities and set up the stage for future functional analyses. Due to the lack of supporting evidence for the suitability of these antibodies in the applications of immunohistochemistry and immunofluorescence, we were not able to address intestinal cell lineage-specific expression patterns of ICK/MAK/MOK proteins in the intestine. Nevertheless, we are the first to report their divergent regional and temporal expression patterns in the intestine and distinct patterns of expression in intestinal cancer.

Compared with MAK and MOK proteins, ICK protein is widely expressed in the intestine along the duodenum-to-colon axis, although the expression level can vary dramatically between different regions in some individuals (Fig. 3 Mouse #3 and #4) for unknown reasons. We speculate that this variation in regional ICK expression may be associated with local luminal nutrition status since food load and nutrient uptake are capable of regulating mucosal growth and growth-related metabolic and signaling pathways [29], [30], [31]. Whether regional ICK expression correlates with the intestinal nutritional status will be an interesting research topic that requires further investigation in the future.

In small intestine, high level of ICK protein remained from postnatal day 1 to day 14, overlapping with the postnatal developmental time-window during which the cellular architecture along the crypt-to-villus axis is established through stem cell activities, as confirmed by the expression pattern of Lgr5, the marker for active intestinal stem cell population. This observation is consistent with the original hypothesis that ICK may play an important role in supporting intestinal epithelial stem cell renewal and lineage allocation during postnatal growth of the intestinal epithelium [1]. Expression of MOK proteins appeared to be restricted to the epithelium of small intestine and peaked at postnatal day 14 when very weak expression of stem cell markers (Lgr5 and Bmi1) was observed, suggesting that MOK is unlikely to play a major role in the regulation of stem cell activities during the postnatal intestinal growth and development. Given a possible role for MOK in the regulation of intestinal differentiation [10], we speculate that MOK may be required for intestinal epithelial cell lineage differentiation and maturation, particularly around postnatal day 14 during intestinal development. MAK protein is predominantly enriched in colon, implicating important roles for MAK activities in the maturation of colonic architecture and functions during postnatal development. Intriguingly, the two intestinal MAK isoforms displayed distinct expression patterns in colon. MAK-2 may have a general “house-keeping” function in colon because its expression was maintained at a high level throughout the postnatal development. In contrast, MAK-1 expression was restricted to the mesenchymal cells and elevated to the level comparable to that of MAK-2 at postnatal day 14, suggesting specific roles for MAK-1 in the colonic mesenchyme during late postnatal development. These spatio-temporal expression profiles of intestinal ICK/MAK/MOK proteins will provide essential information and guidance that facilitate the design of functional analyses and the interpretation of any phenotypic effects in our future loss-of-function or gain-of-function studies.

Previously, we have shown that ICK promotes proliferation and cell cycle progression of intestinal epithelial and colorectal cancer cells *in vitro* [17], consistent with its pro-proliferation role predicted from the specific localization of its mRNA in the intestinal crypt where stem/progenitor cells and rapidly-replicating transient epithelial cells reside [1]. Our data in this report that ICK is significantly over-expressed in human colorectal cancer specimens and intestinal adenomas of the APC^Min/+^ mice as compared with their adjacent normal tissues have provided further support for the role of ICK in promoting intestinal proliferation *in vivo* and implicated ICK as a strong candidate “tumor promoter” gene in colorectal cancer. Further work will be required to correlate the level of ICK expression with the full range of clinical and pathological parameters in colon cancer specimens in order to make the clinical association of ICK over-expression with prognosis and therapy for colon cancer.

To our surprise, even though both ICK and MAK are expressed in colonic epithelial cells, their protein levels were differentially regulated during malignant proliferation in the colonic epithelium, suggesting distinct roles for ICK and MAK in colon cancer. It remains to be determined whether this is true in other tissues such as prostate, stomach, retina, kidney, and testis where both ICK and MAK are abundantly expressed. It is worthy of pointing out that MAK protein is also expressed in prostate epithelial cells and over-expressed in prostate tumor tissues [12]. Therefore, although our data did not reveal any major role for MAK in colon cancer, over-expression of MAK protein could be tightly associated with prostate cancer development.

Our data demonstrate that the protein levels of ICK and MOK can be dynamically regulated in the murine small intestine, albeit in totally opposite directions, during the formation of intestinal adenomas. ICK protein level was significantly up-regulated whereas MOK protein level was markedly down-regulated in intestinal adenomas, suggesting a “tumor promoting” role for ICK and a “tumor suppressor” role for MOK during intestinal tumorigenesis. This result is consistent with a previous report [24] that expression of β-catenin is able to significantly activate the ICK promoter activity since aberrant activation of Wnt/β-catenin pathway triggers adenomatous polyp formation, the initial step toward intestinal neoplasia. Our result is also consistent with the *in vitro* data from HT-29 cells which implicated a pro-differentiation role for intestinal MOK. An intriguing question that yet to be addressed in the future is whether or not there is any cross-talk between ICK and MOK during the regulation of intestinal epithelial proliferation and differentiation.

In conclusion, our studies have demonstrated that intestinal ICK/MAK/MOK proteins exhibit divergent regional and temporal expression patterns. More importantly, their protein levels can be dynamically and differentially regulated during the development of intestinal cancer, suggesting distinct intestinal roles for these phylogenetically-related protein kinases.

## Supporting Information

Figure S1 **The specificities of the ICK, MAK and MOK antibodies used in this study.** Gastrointestinal epithelial cells Colo205 or AGS, as indicated, were treated with either the control lentiviral shRNA or the specific lentiviral shRNA recognizing ICK, MAK, or MOK respectively. Equal amount of total proteins from cell extracts were Western blotted against ICK, MAK, and MOK antibodies respectively. The antibody signals of Hsp90, ERK1/2, or β-Catenin on Western blot were also shown to indicate equal loading and also as controls for non-specific targeting effects.
(TIF)

Figure S2 **Expression of MAK-1 and MAK-2 isoforms in mouse colon.** Tissues extracts were prepared from snap-frozen colon, retina, and testis tissues from young adult mice. Equal amount of total proteins were loaded on Western blot against the MAK antibody that was raised against a common antigen peptide sequence present in all three isoforms of mouse MAK. MAK-1 was first reported in mouse retina; MAK-2 and MAK-3 were first reported in mouse testis. Note that our data indicate weak expression of MAK-1 in testis and MAK-2 in retina as well. MAK-1 and MAK-2, but not MAK-3, were detected in mouse colon.
(TIF)

Figure S3 **Relative abundance of ICK, MAK, and MOK proteins in young adult mouse tissues.** The Western blot signals of ICK/MAK/MOK shown in Figure 2A were quantified by densitometry and normalized against the ERK2 signal. Shown here are the quantitative data indicating relative abundance of ICK, MAK, and MOK proteins in various mouse tissues.
(TIF)

## References

[1] Togawa K, Yan YX, Inomoto T, Slaugenhaupt S, Rustgi AK (2000) Intestinal cell kinase (ICK) localizes to the crypt region and requires a dual phosphorylation site found in map kinases. J Cell Physiol 183: 129–139. 10699974 10.1002/(SICI)1097-4652(200004)183:1<129::AID-JCP15>3.0.CO;2-S

[2] Abe S, Yagi T, Ishiyama S, Hiroe M, Marumo F, et al (1995) Molecular cloning of a novel serine/threonine kinase, MRK, possibly involved in cardiac development. Oncogene 11: 2187–2195. 8570168

[3] Matsushime H, Jinno A, Takagi N, Shibuya M (1990) A novel mammalian protein kinase gene (mak) is highly expressed in testicular germ cells at and after meiosis. Mol Cell Biol 10: 2261–2268. 2183027 10.1128/mcb.10.5.2261-2268.1990 PMC360573

[4] Bladt F, Birchmeier C (1993) Characterization and expression analysis of the murine rck gene: a protein kinase with a potential function in sensory cells. Differentiation 53: 115–122. 8359591 10.1111/j.1432-0436.1993.tb00651.x

[5] Miyata Y, Akashi M, Nishida E (1999) Molecular cloning and characterization of a novel member of the MAP kinase superfamily. Genes Cells 4: 299–309. 10421840 10.1046/j.1365-2443.1999.00261.x

[6] Miyata Y, Nishida E (1999) Distantly related cousins of MAP kinase: biochemical properties and possible physiological functions. Biochem Biophys Res Commun 266: 291–295. 10600495 10.1006/bbrc.1999.1705

[7] Jinno A, Tanaka K, Matsushime H, Haneji T, Shibuya M (1993) Testis-specific mak protein kinase is expressed specifically in the meiotic phase in spermatogenesis and is associated with a 210-kilodalton cellular phosphoprotein. Mol Cell Biol 13: 4146–4156. 8321219 10.1128/mcb.13.7.4146-4156.1993 PMC359964

[8] Ma AH, Xia L, Desai SJ, Boucher DL, Guan Y, et al (2006) Male germ cell-associated kinase, a male-specific kinase regulated by androgen, is a coactivator of androgen receptor in prostate cancer cells. Cancer Res 66: 8439–8447. 16951154 10.1158/0008-5472.CAN-06-1636

[9] Omori Y, Chaya T, Katoh K, Kajimura N, Sato S, et al (2010) Negative regulation of ciliary length by ciliary male germ cell-associated kinase (Mak) is required for retinal photoreceptor survival. Proc Natl Acad Sci U S A 107: 22671–22676. 21148103 10.1073/pnas.1009437108 PMC3012466

[10] Uesaka T, Kageyama N (2004) Cdx2 homeodomain protein regulates the expression of MOK, a member of the mitogen-activated protein kinase superfamily, in the intestinal epithelial cells. FEBS Lett 573: 147–154. 15327990 10.1016/j.febslet.2004.07.070

[11] Fu Z, Schroeder MJ, Shabanowitz J, Kaldis P, Togawa K, et al (2005) Activation of a nuclear Cdc2-related kinase within a mitogen-activated protein kinase-like TDY motif by autophosphorylation and cyclin-dependent protein kinase-activating kinase. Mol Cell Biol 25: 6047–6064. 15988018 10.1128/MCB.25.14.6047-6064.2005 PMC1168834

[12] Wang LY, Kung HJ (2011) Male germ cell-associated kinase is overexpressed in prostate cancer cells and causes mitotic defects via deregulation of APC/C(CDH1). Oncogene. 10.1038/onc.2011.464 PMC3566783 21986944

[13] Fu Z, Larson KA, Chitta RK, Parker SA, Turk BE, et al (2006) Identification of yin-yang regulators and a phosphorylation consensus for male germ cell-associated kinase (MAK)-related kinase. Mol Cell Biol 26: 8639–8654. 16954377 10.1128/MCB.00816-06 PMC1636783

[14] Lahiry P, Wang J, Robinson JF, Turowec JP, Litchfield DW, et al (2009) A multiplex human syndrome implicates a key role for intestinal cell kinase in development of central nervous, skeletal, and endocrine systems. Am J Hum Genet 84: 134–147. 19185282 10.1016/j.ajhg.2008.12.017 PMC2668000

[15] Ozgul RK, Siemiatkowska AM, Yucel D, Myers CA, Collin RW, et al (2011) Exome sequencing and cis-regulatory mapping identify mutations in MAK, a gene encoding a regulator of ciliary length, as a cause of retinitis pigmentosa. Am J Hum Genet 89: 253–264. 21835304 10.1016/j.ajhg.2011.07.005 PMC3155188

[16] Tucker BA, Scheetz TE, Mullins RF, DeLuca AP, Hoffmann JM, et al (2011) Exome sequencing and analysis of induced pluripotent stem cells identify the cilia-related gene male germ cell-associated kinase (MAK) as a cause of retinitis pigmentosa. Proc Natl Acad Sci U S A 108: E569–576. 21825139 10.1073/pnas.1108918108 PMC3161526

[17] Fu Z, Kim J, Vidrich A, Sturgill TW, Cohn SM (2009) Intestinal cell kinase, a MAP kinase-related kinase, regulates proliferation and G1 cell cycle progression of intestinal epithelial cells. Am J Physiol Gastrointest Liver Physiol 297: G632–640. 19696144 10.1152/ajpgi.00066.2009 PMC2763805

[18] Sweetser DA, Birkenmeier EH, Hoppe PC, McKeel DW, Gordon JI (1988) Mechanisms underlying generation of gradients in gene expression within the intestine: an analysis using transgenic mice containing fatty acid binding protein-human growth hormone fusion genes. Genes Dev 2: 1318–1332. 2462524 10.1101/gad.2.10.1318

[19] Whitehead RH, Demmler K, Rockman SP, Watson NK (1999) Clonogenic growth of epithelial cells from normal colonic mucosa from both mice and humans. Gastroenterology 117: 858–865. 10500068 10.1016/s0016-5085(99)70344-6

[20] Shinkai Y, Satoh H, Takeda N, Fukuda M, Chiba E, et al (2002) A testicular germ cell-associated serine-threonine kinase, MAK, is dispensable for sperm formation. Mol Cell Biol 22: 3276–3280. 11971961 10.1128/MCB.22.10.3276-3280.2002 PMC133803

[21] Yan KS, Chia LA, Li X, Ootani A, Su J, et al (2012) The intestinal stem cell markers Bmi1 and Lgr5 identify two functionally distinct populations. Proc Natl Acad Sci U S A 109: 466–471. 22190486 10.1073/pnas.1118857109 PMC3258636

[22] Barker N, van Es JH, Kuipers J, Kujala P, van den Born M, et al (2007) Identification of stem cells in small intestine and colon by marker gene Lgr5. Nature 449: 1003–1007. 17934449 10.1038/nature06196

[23] Sangiorgi E, Capecchi MR (2008) Bmi1 is expressed in vivo in intestinal stem cells. Nat Genet 40: 915–920. 18536716 10.1038/ng.165 PMC2906135

[24] Sturgill TW, Stoddard PB, Cohn SM, Mayo MW (2010) The promoter for intestinal cell kinase is head-to-head with F-Box 9 and contains functional sites for TCF7L2 and FOXA factors. Mol Cancer 9: 104. 20459822 10.1186/1476-4598-9-104 PMC2876993

[25] Oving IM, Clevers HC (2002) Molecular causes of colon cancer. Eur J Clin Invest 32: 448–457. 12059991 10.1046/j.1365-2362.2002.01004.x

[26] Lehner F, Kulik U, Klempnauer J, Borlak J (2010) Inhibition of the liver enriched protein FOXA2 recovers HNF6 activity in human colon carcinoma and liver hepatoma cells. PLoS One 5: e13344. 20967225 10.1371/journal.pone.0013344 PMC2954183

[27] Lehner F, Kulik U, Klempnauer J, Borlak J (2007) The hepatocyte nuclear factor 6 (HNF6) and FOXA2 are key regulators in colorectal liver metastases. FASEB J 21: 1445–1462. 17283222 10.1096/fj.06-6575com

[28] McCart AE, Vickaryous NK, Silver A (2008) Apc mice: models, modifiers and mutants. Pathol Res Pract 204: 479–490. 18538487 10.1016/j.prp.2008.03.004

[29] Jenkins AP, Thompson RP (1994) Enteral nutrition and the small intestine. Gut 35: 1765–1769. 7829017 10.1136/gut.35.12.1765 PMC1375268

[30] Lenaerts K, Sokolovic M, Bouwman FG, Lamers WH, Mariman EC, et al (2006) Starvation induces phase-specific changes in the proteome of mouse small intestine. J Proteome Res 5: 2113–2122. 16944922 10.1021/pr060183+

[31] Raul F, Schleiffer R (1996) Intestinal adaptation to nutritional stress. Proc Nutr Soc 55: 279–289. 8832800 10.1079/pns19960029

