# Supplementary figures and images for: Distinct Expression Patterns of ICK/MAK/MOK Protein Kinases in the Intestine Implicate Functional Diversity
Source: PLoS One. 2013 Nov 7;8(11):e79359. doi: 10.1371/journal.pone.0079359 (PMC3820702; doi:10.1371/journal.pone.0079359)

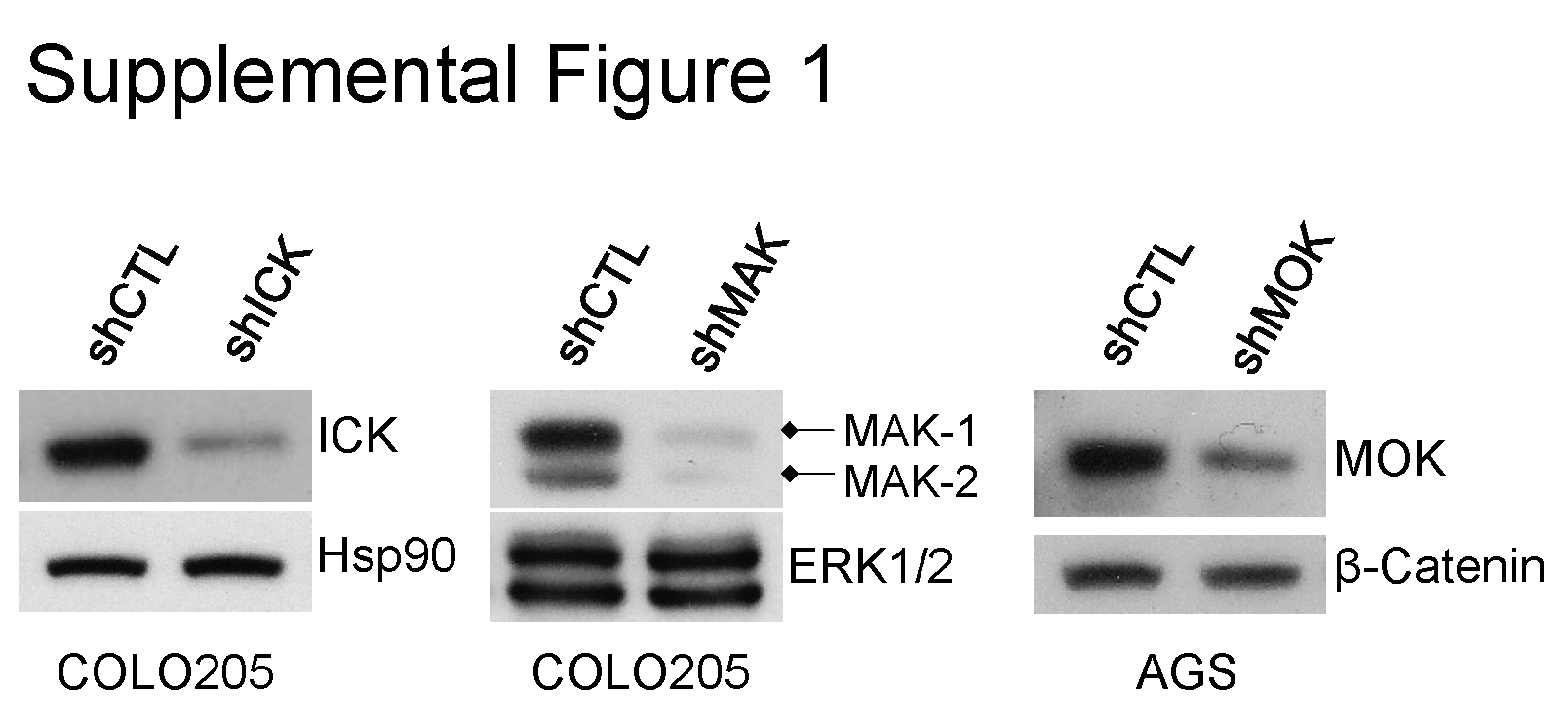

Supplement: Figure S1 — The specificities of the ICK, MAK and MOK antibodies used in this study. Gastrointestinal epithelial cells Colo205 or AGS, as indicated, were treated with either the control lentiviral shRNA or the specific lentiviral shRNA recognizing ICK, MAK, or MOK respectively. Equal amount of total proteins from cell extracts were Western blotted against ICK, MAK, and MOK antibodies respectively. The antibody signals of Hsp90, ERK1/2, or β-Catenin on Western blot were also shown to indicate equal loading and also as controls for non-specific targeting effects. (TIF) [file pone.0079359.s001.tif]

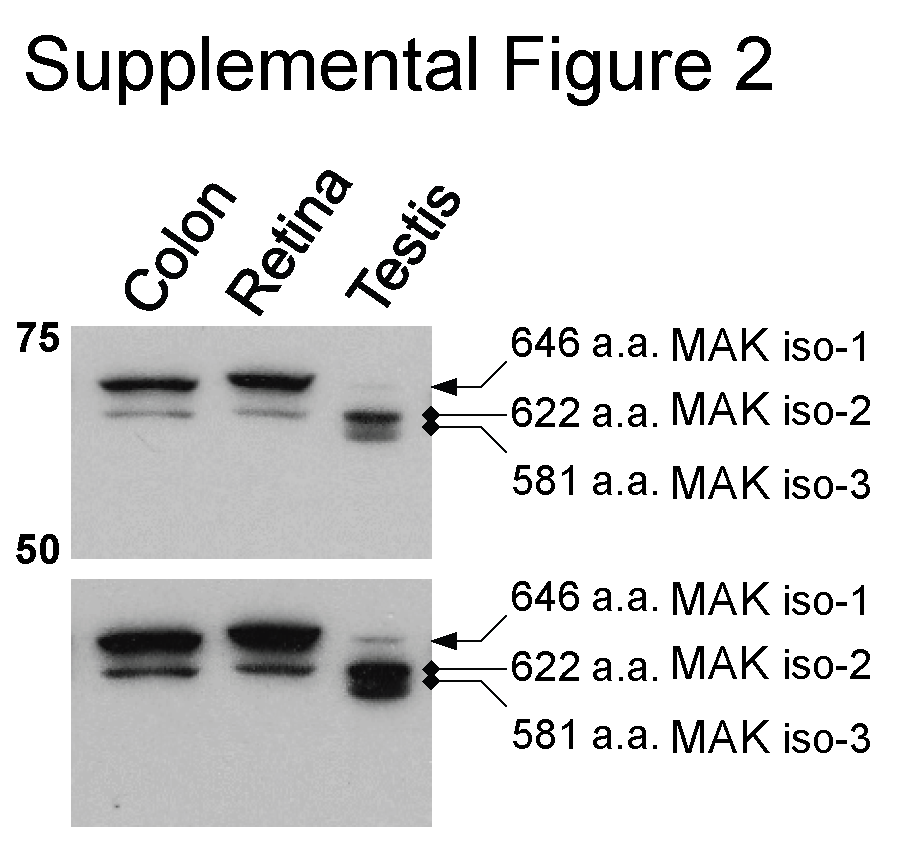

Supplement: Figure S2 — Expression of MAK-1 and MAK-2 isoforms in mouse colon. Tissues extracts were prepared from snap-frozen colon, retina, and testis tissues from young adult mice. Equal amount of total proteins were loaded on Western blot against the MAK antibody that was raised against a common antigen peptide sequence present in all three isoforms of mouse MAK. MAK-1 was first reported in mouse retina; MAK-2 and MAK-3 were first reported in mouse testis. Note that our data indicate weak expression of MAK-1 in testis and MAK-2 in retina as well. MAK-1 and MAK-2, but not MAK-3, were detected in mouse colon. (TIF) [file pone.0079359.s002.tif]

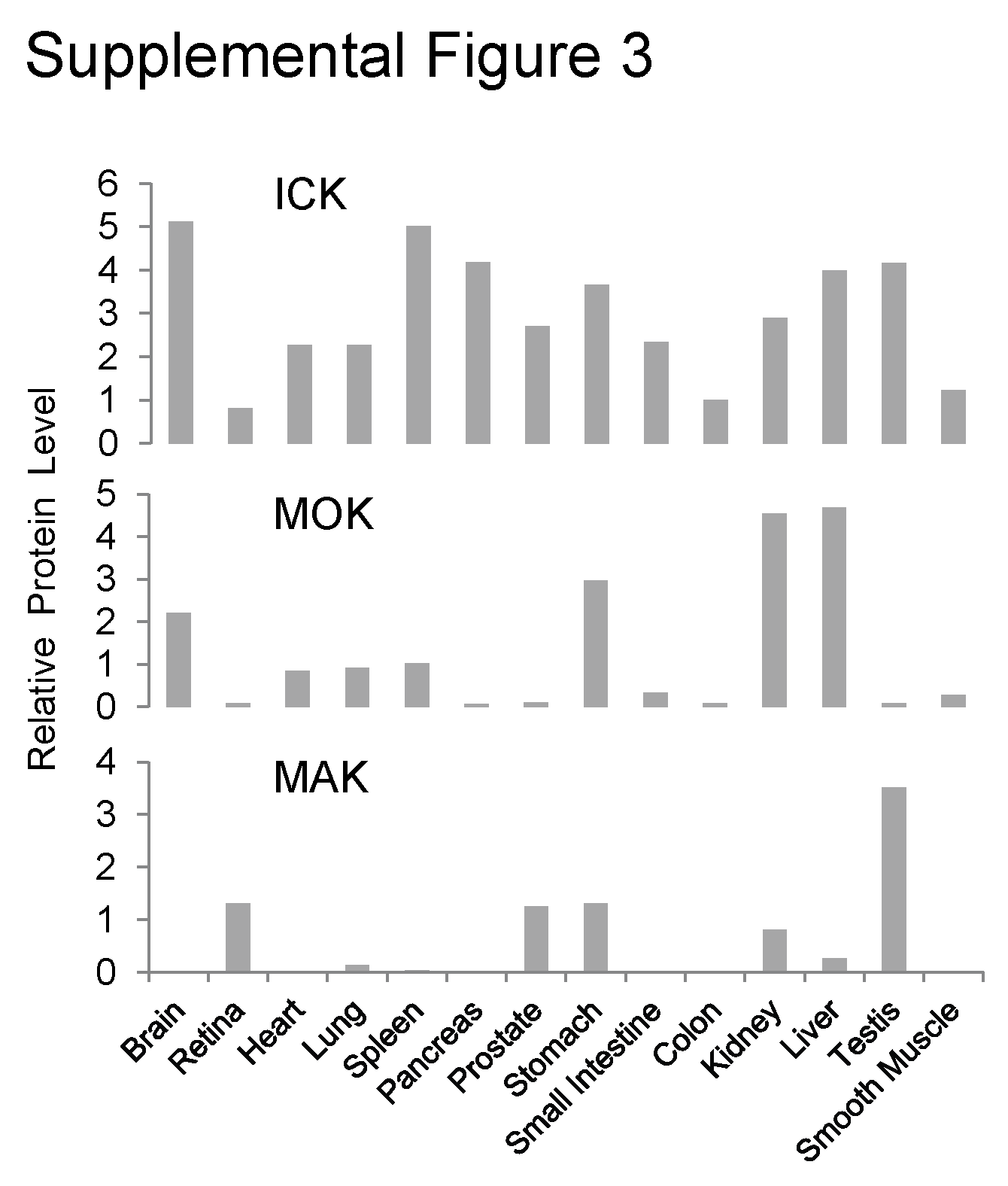

Supplement: Figure S3 — Relative abundance of ICK, MAK, and MOK proteins in young adult mouse tissues. The Western blot signals of ICK/MAK/MOK shown in Figure 2A were quantified by densitometry and normalized against the ERK2 signal. Shown here are the quantitative data indicating relative abundance of ICK, MAK, and MOK proteins in various mouse tissues. (TIF) [file pone.0079359.s003.tif]
